# Obituary

**Published:** 1894-06

**Authors:** 


### Obituary.

From a letter just received from Dr. N. W. Williams, of Nice, France, the word comes that Dr. Henry Shelley, an American dentist, of 33 Boulevard Haussmann, Paris, France, committed suicide, April 12th, shooting himself through the head with a revolver.

Dr. Shelley was about forty-two years of age, and though in comparatively good health had suffered with mental depression for some years. He graduated from the Ohio College of Dental Surgery about the year 1872. He was a man of good ability, but even at that time was subject to times of mental depression, and undoubtedly this peculiarity ultimately became the occasion of his death. He had accumulated a considerble amount of money and property, for which he has no heir in France. His people once lived, we think, and may yet, in Richland county, Ohio. If this notice should come to the knowledge of any of the members of his family it would be to their interest to look after the matter.

Any friends of Dr. Shelley wishing to communicate in reference to the matter can write to Dr. N. W. Williams, of Nice, France, or to Mr. Allain, Attorney, 8 Rue Florentine, Paris, France.

